# Supplementary material for: Neuromodulation Therapy for Chemotherapy-Induced Peripheral Neuropathy: A Systematic Review
Source: Biomedicines. 2022 Aug 7;10(8):1909. doi: 10.3390/biomedicines10081909 (PMC9405804; doi:10.3390/biomedicines10081909)
Supplement: Supplementary file 1 [file biomedicines-10-01909-s001.zip › biomedicines-1817794-supplementary.pdf]

Table S1. Number of results per database.

Database	Results
MEDLINE(R) and Epub Ahead of Print, In-Process, In-Data-Review & Other Non-Indexed Citations	191
Embase	561
Cochrane Central Register of Controlled Trials	65
Cochrane Database of Systematic Reviews	1
Scopus	29

Table S2. Ovid Search Syntax.

**Searches**	**Results**
1. exp Neoplasms/dt [Drug Therapy]	1362312
2. exp Antineoplastic Agents/ae [Adverse Effects]	436933
3. ((("Antineoplastic Agent*" or chemotherap*) adj4 (induced or "adverse effect*" or "adverse event*" or "side effect*")) or (hodgkin* adj1 disease) or adenocarcinoma* or adenoma* or anticarcinogen* or Astrocytoma* or blastoma* or burkitt* or cancer* or carcinogen* or carcinoid* or carcinom* or carcinosarcoma* or cholangiocarcinoma* or chordoma* or "Chronic Myeloproliferative Disorder*" or craniopharyngioma* or ependymoma* or Esthesioneuroblastoma* or germinoma* or "gestational trophoblastic disease*" or Glioblastoma* or glioma* or gonadoblastoma* or hepatoblastoma* or histeocytoma* or histiocytoma* or histiocytos* or leukaemi* or leukemi* or lymphangioma* or lymphangiomyoma* or lymphangiosarcoma* or lymphom* or Macroglobulinemia* or malignan* or melanom* or meningioma* or mesenchymoma* or mesonephroma* or Mesothelioma* or metasta* or "multiple myeloma*" or "Mycosis Fungoide*" or neoplas* or neuroblastoma* or neuroma* or nonmelanoma* or nsclc or oncogen* or oncolog* or ostesarcoma* or Papillomatos* or paraganglioma* or paraneoplas* or pheochromocytoma* or plasmacytoma* or precancerous or retinoblastoma* or Rhabdomyosarcoma* or Sarcoma* or "section 16" or "Szary Syndrome*" or teratocarcinoma* or teratoma* or tumor* or tumour*).ti,ab,kf.	10384202
4. 1 or 2 or 3	10639914
5. exp Peripheral Nervous System Diseases/	244979
6. (Acrodynia or "Alcoholic Neuropath*" or "Alstrom syndrome*" or "Amyloid Neuropath*" or "Brachial Plexus Neuritis" or "Brachial Plexus Neuropath*" or "Carpal Tunnel syndrome*" or "Cauda Equina syndrome*" or Causalgia or "Cervical Rib syndrome*" or "Charcot-Marie-Tooth Disease" or "Complex Regional Pain Syndrome*" or "Congenital Pain Insensitivit*" or "Cubital Tunnel syndrome*" or "Diabetic Neuropath*" or "Familial Dysautonomia*" or "Femoral Neuropath*" or "Giant Axonal Neuropath*" or "Guillain-Barre syndrome*" or "Hand-Arm Vibration syndrome*" or "Hereditary Sensory and Autonomic Neuropath*" or "Hereditary Sensory and Motor Neuropath*" or "Isaacs syndrome*" or "Median Neuropath*" or "Miller Fisher syndrome*" or Mononeuropath* or "Morton Neuroma*" or "Neonatal Brachial Plexus Pals*" or "Nerve Compression Syndrome*" or "Nerve Sheath Neoplasm*" or Neuralgia* or Neuritis or Neurofibroma* or "Neurofibromatosis 1" or neuropath* or "Paraneoplastic Polyneuropath*" or "peripheral nerve disease*" or "Peripheral Nerve Injur*" or "Peripheral Nervous System	612159

	disease*" or "peripheral nervous system disorder*" or "Peripheral Nervous System Neoplasm*" or "peripheral neuropath*" or "Peroneal Neuropath*" or "Piriformis Muscle syndrome*" or "pns disease*" or "POEMS syndrome*" or Polyneuropath* or Polyradiculoneuropath* or Polyradiculopath* or "Pudendal Neuralgia" or "Radial Neuropath*" or Radiculopath* or "Reflex Sympathetic Dystroph*" or "Refsum Disease" or "Sciatic Neuropath*" or Sciatica or "Small Fiber Neuropath*" or "Tangier Disease" or "Tarlov Cyst*" or "Tarsal Tunnel syndrome*" or "Thoracic Outlet syndrome*" or "Tibial Neuropath*" or "Ulnar Nerve Compression Syndromes" or "Ulnar Neuropath*").ti,ab,kf.	
7.	5 or 6	700713
8.	4 and 7	131296
9.	exp electric stimulation therapy/ or spinal cord stimulation/	361489
10.	((("dorsal root" or "dorsal columnor DRGS" or DRG or Electric* or electro or galvano or Transcutaneous* or "spinal cord" or spinal or epidural or "peripheral nerv*" or SCS or PNS) adj5 stimulat*) or electrostimulat* or electrostimulation* or electrostimulus or electrotherap* or "E-stim" or ESTIM or galvanostimulation* or galvanostimulus or neuromodulat* or Neuromodulation or neuromodulatory or neurostimulat* or stimulator*).ti,ab,kf.	441375
11.	9 or 10	728462
12.	8 and 11	1850
13.	exp Antineoplastic Agents/ae [Adverse Effects]	436933
14.	(agent or agents or Antineoplastic* or chemotherap* or drug or drugs or medication* or pharmacotherap*).ti,ab,kf.	8231474
15.	13 or 14	8430055
16.	12 and 15	869
17.	limit 16 to english language [Limit not valid in CDSR; records were retained]	817
18.	limit 16 to no language specified [Limit not valid in CDSR; records were retained]	8
19.	17 or 18	824
20.	limit 19 to (editorial or erratum or note or addresses or autobiography or bibliography or biography or blogs or comment or dictionary or directory or interactive tutorial or interview or lectures or legal cases or legislation or news or newspaper article or overall or patient education handout or periodical index or portraits or published erratum or video-audio media or webcasts) [Limit not valid in CCTR,CDSR,Embase,Ovid MEDLINE(R),Ovid MEDLINE(R) Daily Update,Ovid MEDLINE(R) PubMed not MEDLINE,Ovid MEDLINE(R) In-Process,Ovid MEDLINE(R) Publisher; records were retained]	7
21.	from 20 keep 1	1
22.	(19 not 20) or 21	818

23. remove duplicates from 22	639

Table S3. Scopus Search Syntax.

Searches
1. TITLE-ABS-KEY((("Antineoplastic Agent*" or chemotherap*) W/4 (induced or "adverse effect*" or "adverse event*" or "side effect*")) OR (hodgkin* W/1 disease) OR adenocarcinoma* OR adenoma* OR anticarcinogen* OR Astrocytoma* OR blastoma* OR burkitt* OR cancer* OR carcinogen* OR carcinoid* OR carcinom* OR carcinosarcoma* OR cholangiocarcinoma* OR chordoma* OR "Chronic Myeloproliferative Disorder*" OR craniopharyngioma* OR ependymoma* OR Esthesioneuroblastoma* OR germinoma* OR "gestational trophoblastic disease*" OR Glioblastoma* OR glioma* OR gonadoblastoma* OR hepatoblastoma* OR histeocytoma* OR histiocytoma* OR histiocytos* OR leukaemi* OR leukemi* OR lymphangioma* OR lymphangiomyoma* OR lymphangiosarcoma* OR lymphom* OR Macroglobulinemia* OR malignan* OR melanom* OR meningioma* OR mesenchymoma* OR mesonephroma* OR Mesothelioma* OR metasta* OR "multiple myeloma*" OR "Mycosis Fungoide*" OR neoplas* OR neuroblastoma* OR neuroma* OR nonmelanoma* OR nsclc OR oncogen* OR oncolog* OR ostesarcoma* OR Papillomatos* OR paraganglioma* OR paraneoplas* OR pheochromocytoma* OR plasmacytoma* OR precancerous OR retinoblastoma* OR Rhabdomyosarcoma* OR Sarcoma* OR "section 16" OR "Szary Syndrome*" OR teratocarcinoma* OR teratoma* OR tumor* OR tumour*)
2. TITLE-ABS-KEY(Acrodynia OR "Alcoholic Neuropath*" OR "Alstrom syndrome*" OR "Amyloid Neuropath*" OR "Brachial Plexus Neuritis" OR "Brachial Plexus Neuropath*" OR "Carpal Tunnel syndrome*" OR "Cauda Equina syndrome*" OR Causalgia OR "Cervical Rib syndrome*" OR "Charcot-Marie-Tooth Disease" OR "Complex Regional Pain Syndrome*" OR "Congenital Pain Insensitivit*" OR "Cubital Tunnel syndrome*" OR "Diabetic Neuropath*" OR "Familial Dysautonomia*" OR "Femoral Neuropath*" OR "Giant Axonal Neuropath*" OR "Guillain-Barre syndrome*" OR "Hand-Arm Vibration syndrome*" OR "Hereditary Sensory and Autonomic Neuropath*" OR "Hereditary Sensory and Motor Neuropath*" OR "Isaacs syndrome*" OR "Median Neuropath*" OR "Miller Fisher syndrome*" OR Mononeuropath* OR "Morton Neuroma*" OR "Neonatal Brachial Plexus Pals*" OR "Nerve Compression Syndrome*" OR "Nerve Sheath Neoplasm*" OR Neuralgia* OR Neuritis OR Neurofibroma* OR "Neurofibromatosis 1" OR neuropath* OR "Paraneoplastic Polyneuropath*" OR "peripheral nerve disease*" OR "Peripheral Nerve Injur*" OR "Peripheral Nervous System disease*" OR "peripheral nervous system disorder*" OR "Peripheral Nervous System Neoplasm*" OR "peripheral neuropath*" OR "Peroneal Neuropath*" OR "Piriformis Muscle syndrome*" OR "pns disease*" OR "POEMS syndrome*" OR Polyneuropath* OR Polyradiculoneuropath* OR Polyradiculopath* OR "Pudendal Neuralgia" OR "Radial Neuropath*" OR Radiculopath* OR "Reflex Sympathetic Dystroph*" OR "Refsum Disease" OR "Sciatic Neuropath*" OR Sciatica OR "Small Fiber Neuropath*" OR "Tangier Disease" OR "Tarlov Cyst*" OR "Tarsal Tunnel syndrome*" OR "Thoracic Outlet syndrome*" OR "Tibial Neuropath*" OR "Ulnar Nerve Compression Syndromes" OR "Ulnar Neuropath*")

3.	TITLE-ABS-KEY((("dorsal root" or "dorsal column"or DRGS or DRG or Electric* or electro or galvano or Transcutaneous* or "spinal cord" or spinal or epidural or "peripheral nerv*" or SCS or PNS) W/5 (stimulat*)) OR electrostimulat* OR electrostimulation* OR electrostimulus OR electrotherap* OR "E-stim" OR ESTIM OR galvanostimulation* OR galvanostimulus OR neuromodulat* OR Neuromodulation OR neuromodulatory OR neurostimulat* OR stimulator*)
4.	TITLE-ABS-KEY(agent OR agents OR Antineoplastic* OR chemotherap* OR drug OR drugs OR medication* OR pharmacotherap*)
5.	LANGUAGE(english)
6.	1 and 2 and 3 and 4 and 5
7.	DOCTYPE(ed) OR DOCTYPE(bk) OR DOCTYPE(er) OR DOCTYPE(no) OR DOCTYPE(sh)
8.	6 and not 7
9.	INDEX(embase) OR INDEX(medline) OR PMID(0* OR 1* OR 2* OR 3* OR 4* OR 5* OR 6* OR 7* OR 8* OR 9*)
10.	8 and not 9
